# Relationships between children-related factors, basic psychological need satisfaction, and multiple happiness among urban empty-nesters in China: a structural equation modeling

**DOI:** 10.1186/s12877-022-03640-0

**Published:** 2022-12-01

**Authors:** Yang Yu-ting, Yao Miao, Yang Yong-wei, Ye Qiong, Lin Ting

**Affiliations:** 1 Nursing Department, the First Affiliated Hospital of Fujian Medical University, Fuzhou, Fujian China; 2 The School of Nursing, Fujian Medical University, Fuzhou, Fujian China; 3 Ningde Municipal Hospital of Ningde Normal University, Ningde, Fujian China

**Keywords:** China, Empty-nester, Basic psychological need satisfaction, Multiple happiness, Urban

## Abstract

**Background:**

Aging and empty-nesting in China are becoming more and more serious. Empty-nesters refer to older adults who were not living with their children, were living alone, or were living independently with their spouses. The mental health of empty-nesters needs timely attention. Satisfying the needs of older adults is primarily dependent on their children. Therefore, this study aimed to explore relationships between children-related factors, basic psychological need satisfaction, and multiple happiness among urban empty-nesters in China.

**Methods:**

This study employs the Children-related Factors Questionnaire (CFQ), the Chinese Version of the Basic Psychological Need Satisfaction (BPNS) Scale, and the Multiple Happiness Questionnaire (MHQ) to explore children-related factors, basic psychological need satisfaction and multiple happiness of urban empty-nesters in Fuzhou, China. A total of 507 samples were recruited by cluster sampling.

**Results:**

Factors that affect BPNS include physical condition, the number of children, satisfaction with children's marriage, employment and income, retirement, gender, monthly personal income, and self-care have statistical differences (*P* < 0.05). Factors that affect MHQ include physical condition, the number of children, satisfaction with children's marriage, employment and income, gender, monthly personal income, living status, and self-care have statistical significances (*P* < 0.05). Structural equation model results showed that BPNS directly impacted MHQ, and factors about children indirectly affect MHQ through BPNS. The mediating role of BPNS in the relationship between children-related factors and MHQ.

**Conclusion:**

We should encourage our children to spend more time with the empty-nesters and communicate with them, which could help them relieve their negative emotions, satisfy their psychological needs, and improve their well-being.

**Trial registration:**

Reg date: 20/08/2021; No. ChiCTR2100050175.

**Supplementary Information:**

The online version contains supplementary material available at 10.1186/s12877-022-03640-0.

## Introduction

Globally, there were 703 million persons aged 65 or over in 2019. East and South-East Asia have the largest aged population, approximately 261 million [1]. Aging and empty nesting in China are also becoming more and more serious. The seventh census [2] showed that at the beginning of November 2020, the number of people aged 60 and over in China was 264 million, accounting for 18.70% of the total population. Affected by national fertility policy and changes in public fertility concepts, many Chinese people face serious "empty-nest" problems. They live independently or alone without living with children. The proportion of empty-nest and single-living aged families in China has been increasing [3]. Empty-nesters accounted for 51.90% of the total number of older adults in 2015. Among them, older adults living alone accounted for 10.00% of the total number of older adults, and those living with their spouses accounted for 41.90% of the total [4]. By 2030, this proportion will reach 90%, which shows there will be more than 200 million empty-nesters in China [5].

The health status of empty-nesters has been a broad concern in the academic circle. But physical health has always been a higher concern than mental health [6]. However, empty-nest life often causes passive mental health [7]. Empty-nest living would produce endless psychological problems, such as loneliness [8], Anxiety, and depression [9]. The prevalence of mental diseases would cause a negative quality of life and hinder the generation and improvement of happiness [10]. Therefore, to improve the quality of life and life satisfaction of our nationals, we need to pay more attention to the well-being of the increasingly large number of older adults, especially empty-nesters. Sociodemographic factors about gender, age, education, self-assessed physical health, marriage, income, and chronic diseases [11–13] would affect empty-nesters' happiness. The effects of these factors in various regions of China are different. A study [11] showed rural empty-nesters who are low-age, female, married, educated, with spouses, good income, and self-assessed physical health had high subjective well-being (SWB) in Wenzhou, China. In an inland mountainous county, Hubei, China, compared with non-empty-nesters, empty-nesters have lower income, a higher prevalence of chronic diseases, and lower life satisfaction [12]. However, life satisfaction of the rural left-behind older adults is generally higher, and the level is higher in males than in females in Sichuan, China [13].

### Children-related factors

Compared with non-empty-nesters, children-related factors are a particular issue affecting well-being of empty-nesters [14]. In general, the life satisfaction of older adults reflects their well-being [15]. Research shows the relationship between children and older adults' life satisfaction is mainly based on two aspects, which are a relationship between the objective characteristics of children and the life satisfaction of older adults, and the effect of children's support on older adults' subjective life perceptions [13]. In terms of objective factors, the number of children and distance between parents and children would affect the life satisfaction of older adults [16]. Older adults with more children have higher life satisfaction [17]. Compared with empty-nesters without children, people who have children and frequently meet their children reported high SWB [11]. Parents whose children live far away have low life satisfaction [18]. In terms of children's support, filial piety was not only an eventful form of support for older adults' spiritual life but a significant source of their emotional needs. Empty-nesters close to their children will be more likely to enjoy life, make friends, develop their interests, and keep a better psychological state [19]. However, the acquired social support of the empty-nesters is limited. The support and care of family members, especially their children, are particularly pivotal [20]. Children's support to parents can reflect older parents' quality of life and life satisfaction [21]. Parents' quality of life decreased when a child became unemployed and increased when a child started living with a partner [14]. Besides that, Li et al. [22] found that the higher the income level of the children, the higher the parents' life satisfaction. Older parents' satisfaction with their children's marriage, employment, and income directly affects their happiness [21]. Therefore, this study turns children's support to parents into three more specific aspects: parents' satisfaction with children's marriage, employment, and income. Based on the above research, this study will explore children-related factors that affect the well-being of older parents in two aspects: objective characteristics (the number of children and distance between parents and children) and children's support (satisfaction with children's marriage, employment, and income).

### Well-being

As a significant evaluation index of mental health, well-being has also become a point in geriatric psychology [23]. The research on well-being in modern psychology originates from two philosophical viewpoints, which are hedonic ideas centering on experiences of personal happiness, and eudaemonic on the meaning of life and self-realization. The ideas produce two different paradigms: subjective well-being (SWB) and psychological well-being (PWB) [24]. SWB originates in the hedonic ideas, which define well-being in terms of pleasure vs. pain. It usually refers to the results of the total evaluation of the quality of life by the evaluator according to self-defined criteria [25]. Now, researchers on SWB at home and abroad are relatively mature. Definition and measurement of SWB have reached a consensus. The classic three evaluation indicators of SWB are positive affect, negative affect, and life satisfaction [26]. PWB starts from theory, constructs the structure of well-being, begins at the eudaemonic ideas, which define well-being in terms of the degree to which a person is fully functioning, and guides the development of well-being measurement. It believes that happiness is not only an emotional experience but also emphasizes realizing personal self-improvement, self-achievement, and self-potential [27, 28].

### Self-determination theory

Self-Determination Theory (SDT) was proposed in the 1980s by two American psychologists, Deci and Ryan [29], which showed that human beings have three basic psychological needs autonomy, competence, and relatedness [30]. Autonomy manifests in actions that gain self-approval or are consistent with one's values. Competence refers to feeling efficient and mastery, and people can function effectively in critical life situations. Relatedness is being able to feel connected to others. People would have a sense of belonging and be valued members of a social group [28]. Three basic psychological needs satisfaction is interrelated.

Deci and Ryan [31] presented that eudaemonic living fosters well-being because it provides the satisfaction of people’s most fundamental needs. The core assumption of SDT is that, regardless of the conditional factors, satisfying basic psychological needs will increase the health level, while unsatisfying them will reduce the health level [28]. Given the roles that basic needs play in reflecting the extent to which individuals experience satisfaction or frustration from their activities and environments, need satisfaction and need frustration presumably serve as a bridge connecting ways of living and well-being [32]. Three basic needs as basic nutrients are decisive for optimal experience and well-being in daily life, and satisfying these three needs can promote health and well-being [30]. The reason is people who pursue eudaimonia tend to engage in activities that tap into their deep psychological needs, which further contribute to their well-being [33]. For example, people who pursue intrinsic life goals such as belonging and personal growth experience increased needs satisfaction compared to those who pursue extrinsic life goals such as fame and wealth [34]. Given the robust evidence showing the positive link between need satisfaction and well-being, the mediating role of need satisfaction should be established [35].

### Basic psychological need satisfaction and well-being

Under the guidance of self-determination theory, scholars from different countries have conducted surveys on older adults in various regions and proved a positive correlation between basic psychological need satisfaction and well-being. Based on the self-determination theory, Custers et al. [36] conducted a questionnaire survey on 88 middle-aged and older adults in Dutch nursing homes. They found that if meeting the needs of middle-aged and older adults, their depression would reduce, and life satisfaction and subjective well-being would increase in the care relationship. Ferrand et al. [37] also conducted a questionnaire survey on 100 older adults in French nursing homes based on the theory of self-determination. The study showed that autonomy and relatedness satisfaction was positively associated with well-being. Kasser and Ryan [38] explored the effects of autonomy and relatedness on well-being of 50 residents at a nursing home in upstate New York via interviews and surveys. It also found that satisfying these two psychological needs were positively correlated with well-being, and in both they were happiness can be forward predicted. Haiyan et al. [39] investigated 289 middle-aged and older patients with coronary heart disease in Weifang, Shandong Province. There is a positive correlation between basic psychological need satisfaction and subjective well-being. Tang et al. [40] conducted a questionnaire survey on 464 older adults in China and 129 older adults in France. Autonomy, competence, and relatedness need satisfaction can forward predict the psychological well-being of older adults in China and France, and the impact of basic psychological need satisfaction on the psychological well-being of older adults has cross-cultural universality. In short, SDT is one of the classic theories for exploring positive psychology that scholars at home and abroad agree with.

Most studies exploring the relationship between basic psychological need satisfaction and well-being have focused on Europe, Canada, and the U. S. [41]. Studies on Chinese populations are still relatively rare. Although Haiyan et al. [39] showed that basic psychological need satisfaction and subjective well-being of Chinese older adults are positively correlated. Tang et al. [40] showed that basic psychological need satisfaction of Chinese older adults positively correlates with psychological well-being. However, there is currently no article that can demonstrate that meeting the basic psychological needs of Chinese older adults can predict subjective and psychological well-being together. As well as in the context of aging and the increasingly severe empty-nest phenomenon, there are fewer empirical studies on the relationship between children-related factors, basic psychological need satisfaction, and happiness of empty-nesters. Therefore, this study aimed to expand the sample of basic psychological needs satisfaction and well-being research among older adults in various regions in China, and explore the relationships between children-related factors, basic psychological need satisfaction, and multiple happiness of empty-nesters.

Our research aims are as follows: (a) to describe the status quo of basic psychological need satisfaction and multiple happiness; (b) to analyze sociodemographic and children-related factors of basic psychological need satisfaction; (c) to analyze sociodemographic and children-related factors of multiple happiness; (d) to explore relationships between children-related factors, basic psychological need satisfaction, and multiple happiness.

Based on existing literature, we make the following hypothesis: (H1) empty-nesters with many children, living close to children and high satisfaction with their children's employment, income, and marriage have higher BPNS; (H2) empty-nesters with many children, living close to children and high satisfaction with their children's employment, income, and marriage have more happiness; (H3) BPNS plays a mediating role, and children-related factors indirectly affect happiness through BPNS.

## Methods

### Study site

Fuzhou is a city in southern China and the capital of Fujian Province. It is one of the central cities of the economic zone on the west side of the Chinese Taiwan Straits and is a famous historical and cultural city. The city has 6 districts, 6 counties, and 1 county-level city with a total area of 1,1968 square kilometers and a built-up area of 416 square kilometers. In 2021, the permanent population is 8.42 million, and the urbanization rate is 73% [42]. The seventh census shows that Fuzhou has 1,389,989 people aged 60 and above, accounting for 16.76%, of which 971,594 people are 65 years old and above, accounting for 11.72%. Compared with the sixth national census in 2010, the proportion of the population aged 60 and above in Fuzhou increased by 4.67 percentage points, and the proportion of the population aged 65 and above increased by 3.51 percentage points [43]. In 2010, there were 1.09 million urban and rural empty-nesters aged 60 and above in Fujian Province, accounting for about 25.8% of aged population; in 2015, it exceeded 1.3 million. Now, the number of older adults in Fujian Province is rising, and Fuzhou's aging and empty-nest forms are also severe [44].

### Study design and participants

From April to July 2021, we conducted a population-based cross-sectional study in Fuzhou, China. We adopted multi-stage random cluster sampling to get a representative sample of urban empty-nesters. We randomly selected two districts in Fuzhou, one street in each district, and four residential areas in each street. First, according to the order of six districts on the government’s website, each district in Fuzhou was numbered. Second, two districts in Fuzhou were selected using the Research Randomizer Website (https://www.randomizer.org/), which can draw random samples scientifically. Third, one street was randomly selected from each district by this method. Fourth, we used the same method to extract four residential areas from each street. The eight residential areas finally selected are four residential areas under the jurisdiction of Shanghai Street in Taijiang District and four residential areas under the jurisdiction of Wenquan Street in Gulou District. We invited all empty-nesters who meet the following inclusion criteria, agree to accept the study and come from these eight residential areas. Inclusion criteria included age older than 60-year-old, having children alive but living alone or with a couple, living in urban residential areas for 12 months or more, ability to express and comprehend simple Chinese characters, and willingness to complete questionnaires. Exclusion criteria were hearing impairment, speech impairment, severe cognitive impairment, mental illness, and other severe or terminal diseases. Researchers judged whether participants met the inclusion criteria and whether to exclude them. Researchers would ask the following questions when touching older adults: (1) How old are you? (2) Who lives with you? (3) How long have you lived in the urban residential areas of Fuzhou? (4) Are you currently taking certain drugs? (5) Do you currently have diagnosed diseases? Through these questions, the researchers judged whether older adults had normal hearing and speech expression ability, whether they had severe diseases, and were suitable for participating in this study.

All participants were informed of the study procedure upon their recruitment. After obtaining written consent, participants were interviewed by trained study interviewers. Of this sample, 552 participants were invited to answer a standardized questionnaire, and 507 participants completed the study. The response rate of questionnaires was 91.85% (507/552). This study was reviewed and approved by the Ethics Review Committee of Fujian Medical University (IRB Ref. No.: 2021/00,096).

### Measurements

#### Participants’ demographic characteristics questionnaire

The questionnaire was compiled by researchers according to the study purpose and relevant literature, including gender, age, education, marriage, living with whom, whether retirement or not, (pre-retirement) occupation, income source, personal monthly income, self-rated health, among others.

#### Children-related factors questionnaire (CFQ)

Children-related Factors Questionnaire (CFQ) was a 5-item questionnaire compiled by researchers according to our study purpose and relevant literature, including objective characteristics (the number of children and distance between parents and children) and children's support (satisfaction with children's marriage, employment, and income). "The number of children" includes two options with one child or two above. "The distance between parents and children" is two options that at least a child or without children lives in the same city. Objective characteristics are not used for reliability and validity analysis. Satisfaction with children's marriage, employment, and income is provided on a 5-point Likert scale ranging from 1 (strongly dissatisfied) to 5 (strongly satisfied). If the answer is dissatisfied or strongly dissatisfied, the response scores 1. If the answer is general, the response scores 2. If the answer is satisfied or strongly satisfied, the response scores 3. The higher the score, the higher satisfaction with children's marriage, employment, and income. In this study, the total *Cronbach's* *α* coefficient is 0.739, and split-half reliability is 0.705. Independent samples *t*-test and one-way ANOVA were used to distinguish differences in three satisfaction scores by the empty-nesters with different demographic characteristics (discriminant validity) [45]. Discriminant validity showed that satisfaction score with children's marriages was statistically significant in physical health (*F* = 3.561, *P* = 0.029). Satisfaction score with children's employment was statistically significant in personal monthly income (*F* = 2.889, *P* = 0.035) and physical health (*F* = 4.266, *P* = 0.015). Satisfaction score with children's income was statistically significant in different age groups (*F* = 3.430, *P* = 0.033), personal monthly income (*F* = 4.098, *P* = 0.007), and physical health (*F* = 3.626, *P* = 0.027).

#### Multiple happiness questionnaire (MHQ) [46]

**Fig. 1:**
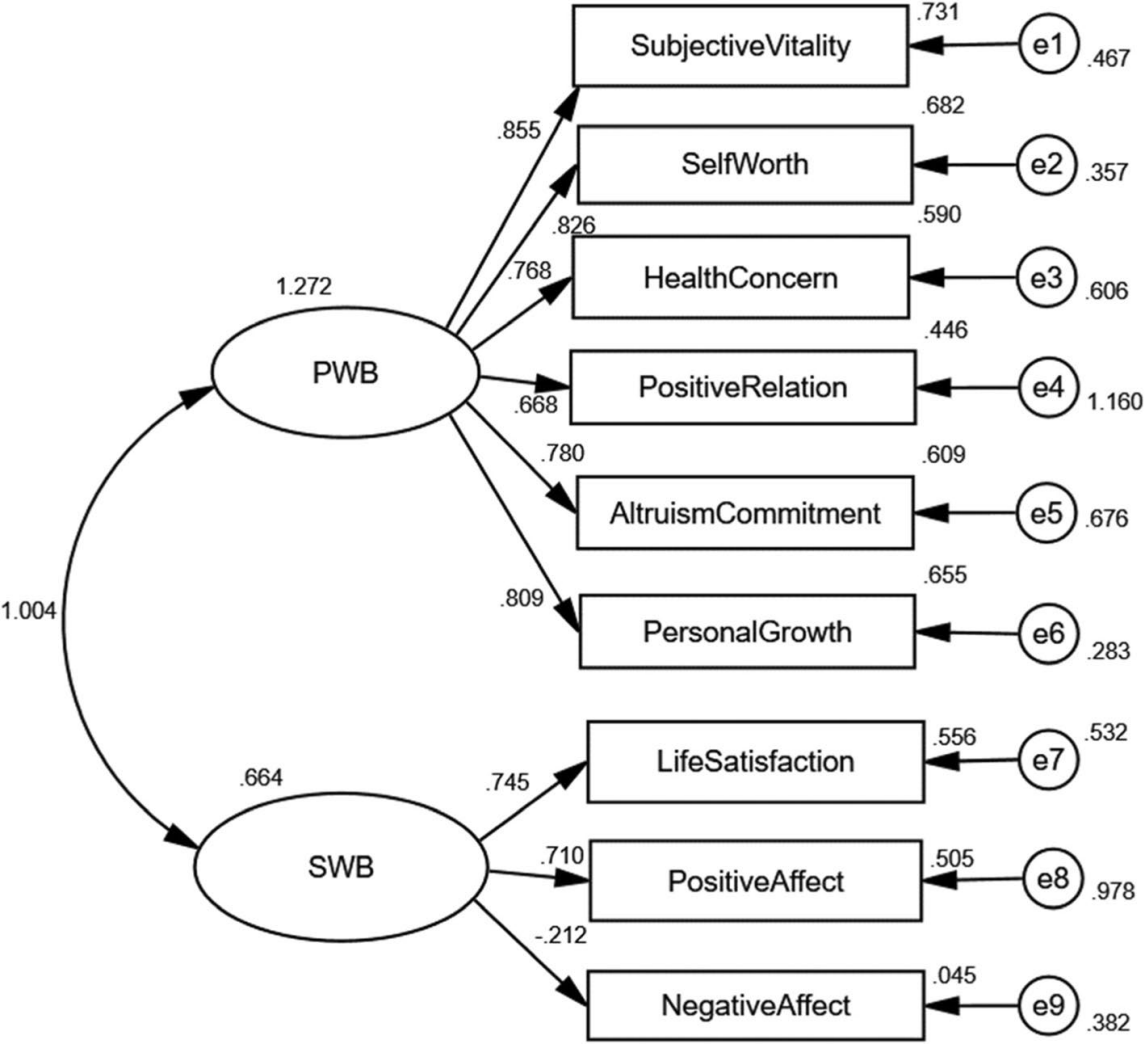
Confirmatory factor analysis standardized correlation diagram (*n* = 507)

Multiple Happiness Questionnaire (MHQ) [24] is a 51-item simplified Chinese scale with 3-part, including SWB, PWB, and happiness index (HI), and 9-dimension, including life satisfaction (5 items), positive affect (6 items), negative affect (6 items), subjective vitality (6 items), self-worth (5 items), health concern (5 items), positive relation (3 items), altruism commitment (5 items), and personal growth (9 items). Firstly, SWB contains the first three dimensions. Responses are provided on a 7-point Likert scale ranging from 1 (strongly inconsistent) to 7 (strongly consistent), which is ≥ 5 is regarded as a high score, and ≤ 3 is a low score, and the lower the negative affective dimension score, the better [47]. Secondly, PWB contains the last six dimensions, which of item 12 and item 14 are reverse-scored, which are also a 7-point Likert scale ranging from 1 (no time) to 7 (all-time), which is ≥ 5 is regarded as a high score, and ≤ 3 is a low score. Thirdly, the happiness index only has a 9-point question, which assesses total happiness. A higher score indicates better happiness. Fourth, the total *Cronbach's α* coefficient is 0.909, nine subscales *Cronbach's α* coefficients are 0.692 ~ 0.912, split-half reliabilities are 0.645 ~ 0.911, and construct validity is up to standard in older adults [48]. The scale is appropriate for Chinese older adults to assess happiness. Finally, PWB's total score is six dimensions' total score is divided by the item number and multiplied by six. SWB's total score is life satisfaction adding with positive affect, minus negative effect, whose total score is divided by item number and multiplied by three. In this study, the total *Cronbach's α* coefficient is 0.962, nine subscales are 0.618 ~ 0.958, and split-half reliabilities are 0.621 ~ 0.948, indicating good reliability. Construct validity was examined by confirmatory factor analysis using the IBM SPSS Amos version 24.0 (IBM Corporation, Armonk, NY, United States) to obtain a two-factor structural equation model. Figure 1 shows that the standardized factor loadings, ranging from -0.212 to 0.855, were statistically significant in the two-factor model, which is similar to the original author's result [24].

#### Chinese version of the basic psychological need satisfaction (BPNS) Scale [46]

Basic Psychological Need Satisfaction (BPNS) Scale [49] translated into simplified Chinese [50] was used in our study. The Chinese version of BPNS comprises 9-item grouped into 3 subscales, with 3-item each for assessing one of the needs, including Autonomy, Competence, and Relatedness. Responses are provided on a 7-point Likert scale ranging from 1 (strongly disagree) to 7 (strongly agree). The average score of 3-item contained in each dimension has expressed each need satisfaction. The average score of 9-item has expressed the total need satisfaction. The higher the score, the higher the satisfaction. The scale is appropriate for Chinese older adults to assess BPNS [46], which total *Cronbach's α* coefficient is 0.877, and the three subscales are 0.826 (autonomy), 0.807 (competence), and 0.847 (relatedness), indicating good reliability. And construct validity and calibration validity are also good in Chinese older adults. In this study, the total *Cronbach's α* coefficient is 0.889, and the three subscales are 0.852 (autonomy), 0.834 (competence), and 0.872 (relatedness). Convergent validity of the Chinese Version of the BPNS was examined with the MHQ using the Pearson correlation coefficient [51], which is 0.729 (*P* < 0.05), indicating that basic psychological need satisfaction is correlated positively with happiness.

### Data collection

Before the collection stage, the Ethical Review Committee of a medical college in Fujian approved the study. Investigators consisted of 3 graduate students and several undergraduates in the college. In the preparation stage, investigators had been uniformly trained, emphasized the application of uniform instructions, and underwent 1 or 2 pre-investigation pieces of training. Before the formal investigation, investigators informed participants of study's purpose, significance, usefulness, privacy rights, etc. Researchers notified rights of each respondent, including refusing to participate and answering any question, both initially and during the study. They signed informed consent, then conducted one-to-one questionnaire surveys.

Formal investigation stage, each questionnaire was conducted through face-to-face interviews between trained investigators and respondents. Investigators read every item in the questionnaire in a neutral, unbiased manner as most of the respondents were old and had relatively low education. They filled out the questionnaire accordingly after ensuring that the respondents understood the question and gave their answers independently, and immediately checked it after finishing the questionnaire. If questionnaires were missing items or had obvious logical errors, participants filled in or modified them on the spot. After checking again, investigators withdraw the questionnaire. Finally, participants were usually able to fill in questionnaires within 30 ~ 40 min.

After collecting data, researchers used rigorous numbers instead of names to mark questionnaires. Questionnaires and their identity information were preserved separately, and numbers corresponded with participants' identity information. All researchers were required to ensure the confidentiality of participants' identities. Researchers could not disclose their identity information to anyone without participant's permission. Personal information was kept in a locked files cabinet and was only opened and browsed by researchers. When necessary, members of government management departments or ethics committees could consult the identity information in the research unit according to regulations.

### Data analysis

IBM SPSS Statistics version 26.0 (IBM Corp., Armonk, NY, USA) and IBM SPSS Amos version 24.0 were used for data filing and statistical analysis, including statistical description and statistical inference. (1) Statistical description: Quantitative data and normally distributed data (dimensions of BPNS) were represented by the mean and standard deviation (‾*x* ± *s*). Those with non-normal distribution were expressed as median (*M*) and interquartile range (*QR*). Count data (socio-demographic variables) were expressed by frequency (*n*) and constituent ratio (*%*). (2) Statistical inference: (a) Analysis of influencing factors for basic psychological need satisfaction: dimensions scores and total score of BPNS were dependent variables. Socio-demographic variables were independent variables to conduct multiple linear regression analysis (Stepwise) filter variables. (b) Analysis of influencing factors for multiple happiness: dimensions scores of MHQ were dependent variables. Socio-demographic variables were independent variables to conduct multiple linear regression analysis (Stepwise) filter variables. (c) Correlation analysis between BPNS and multiple happiness: Pearson correlation analysis was used to analyze normality data, and Spearman correlation analysis was used for non-normal data. (d) Predictive analysis of BPNS on multiple happiness: taking HI, PWB, and SWB as dependent variables, and autonomy, competence, and relatedness as independent variables, multiple linear regression analysis (Stepwise) was conducted. The inclusion criterion was 0.05, and the exclusion criterion was 0.10. *P* < 0.05 means the difference is statistically significant. (e) The structural equation model was used to quantify the hypothesized association between children-related factors, BPNS, and MHQ. Overall model goodness of fit was assessed using χ^2^/*df* ≤ 5.0, goodness-of-fit index (GFI) > 0.90, adjusted goodness-of-fit index (AGFI) > 0.90, comparative fit index (CFI) > 0.90, normed fit index (NFI) > 0.90, Tucker-Lewis index (TLI) > 0.90, standardized root mean square residual (SRMR) < 0.08, and root means square error of approximation (RMSEA) < 0.08 [52, 53].

## Results

### Demographics

Sociodemographic characteristics of 507 persons are shown in Table 1.

**Table 1:** Sociodemographic characteristics of urban empty-nesters

Variable	Categories	*n* (*%*)
Age, year (Range 60 ~ 95)	60 ~ 69	133(26.20)
	70 ~ 79	222(43.80)
	≥ 80	152(30.00)
Gender	Male	313(61.70)
	Female	194(38.30)
Education	Primary school or below	66(13.00)
	Junior high school	110(21.70)
	Senior high school	148(29.20)
	Junior college or above	183(36.10)
Marital status	Married	451(89.00)
	Other	56(11.00)
Living status	Living alone	81(16.00)
	Living with spouse	426(84.00)
Retirement	Yes	467(92.10)
	No	40(7.90)
Pre-retirement occupation	Civil servants	74(14.60)
	Institutional staff	120(23.70)
	Corporate staff	211(41.60)
	Farmers and other occupations	102(20.10)
Main source of income	At least pension	465(91.70)
	Other	42(8.30)
Monthly personal income (RMB)	< 1000	17(3.40)
	1000 ~ 2000	84(16.60)
	2000 ~ 4000	216(42.60)
	≥ 4000	190(37.50)
Types of medical insurance	Urban employee medical insurance	455(89.74)
	Urban and rural residents' medical insurance	52(10.26)
Physical condition	Good	225(44.40)
	General	228(45.00)
	Poor	54(10.70)
Number of chronic diseases	None	152(30.00)
	1 ~ 2	307(60.60)
	≥ 3	48(9.50)
Self-care	Completely self-care	482(95.10)
	Partially self-care	25(4.90)
The number of children	1	235(46.40)
	≥ 2	272(53.60)
The distance between parents and children	At least a child lives in the same city	426(84.00)
	Without children lives in the same city	81(16.00)
Satisfaction with children's marriage	Satisfaction	361(71.20)
	General	117(23.10)
	Dissatisfaction	29(5.70)
Satisfaction with children's employment	Satisfaction	386(76.10)
	General	106(20.90)
	Dissatisfaction	15(3.00)
Satisfaction with children's personal income	Satisfaction	366(72.20)
	General	125(24.70)
	Dissatisfaction	16(3.20)

### Status quo of basic psychological need satisfaction and multiple happiness

The total score and dimensions scores of BPNS and dimensions scores of multiple happiness are shown in Table 2. The overall basic psychological need satisfaction of empty-nesters in Fuzhou was relatively high, and the relatedness score was the highest among the three dimensions. The whole psychological well-being and subjective well-being of empty-nesters in Fuzhou were relatively high, and 73.57% of empty-nesters' self-reported HI was above the middle level.

**Table 2:** Scores of basic psychological need satisfaction (*n* = 507, ‾*x* ± *s*)

Scale	Dimensions	Scores
Basic psychological need satisfaction	Autonomy	5.63 ± 1.14
	Competence	5.27 ± 1.26
	Relatedness	5.68 ± 1.13
	Total scale	5.52 ± 0.98
Multiple happiness	HI	6.81 ± 1.44
	PWB	31.89 ± 5.79
	SWB	8.82 ± 2.38

### Sociodemographic and children-related factors of basic psychological need satisfaction

According to the BPNS, each dimension is equally divided into dependent variable, and all socio-demographics are used as independent variables. Assignment is shown in Table 3. Multiple linear regression analysis results are shown in Table 4.

**Table 3:** Multiple Linear Regression Analysis Assignment

Variable	Variable name	Assignment
Dependent variable	Dimensions scores and total score of BPNS	Continuous numeric variable
	Dimensions scores of MHQ	Continuous numeric variable
Independent variable	Age	1 = 60 ~ 69, 2 = 70 ~ 79, 3 = ≥ 80
	Gender	1 = Male, 0 = Female
	Education	1 = Primary school or below, 2 = Junior high school, 3 = Senior high school, 4 = Junior college or above
	Marital status	1 = Married, 0 = Other
	Living status	1 = Living alone, 0 = Living with spouse
	Retirement	1 = Yes, 0 = No
	Pre-retirement occupation	(1, 0, 0) = Civil servants, (0, 1, 0) = Institutional staff, (0, 0, 1) = Corporate staff, (0, 0, 0) = Farmers and other occupations
	Main source of income	1 = At least pension, 0 = Other
	Monthly personal income (RMB)	1 = < 1000, 2 = 1000 ~ 2000, 3 = 2000 ~ 4000, 4 = ≥ 4000
	Types of medical insurance	1 = Urban employee medical insurance, 0 = Urban and rural residents' medical insurance
	Physical condition	1 = Good, 2 = General, 3 = Poor
	Number of chronic diseases	1 = None, 2 = 1 ~ 2, 3 = ≥ 3
	Self-care	1 = Completely self-care, 0 = Partly self-care
	The number of children	1 = one child, 0 = ≥ 2 children
	The distance between parents and children	1 = At least 1 child live in the same city, 0 = Do not live in the same city
	Satisfaction with children's marriage	1 = Satisfaction, 2 = General, 3 = Dissatisfaction
	Satisfaction with children's employment	1 = Satisfaction, 2 = General, 3 = Dissatisfaction
	Satisfaction with children's personal income	1 = Satisfaction, 2 = General, 3 = Dissatisfaction

**Table 4:** Multiple linear regression analysis of influencing factors about basic psychological need satisfaction in empty-nesters

Dependent variable	Independent variable	*B-value*	*S.E.-value*	*β-value*	*t-value*	*R-value*	*F-value*
Autonomy	Constant	6.445	0.250	-	25.761*	0.316	11.154*
	Physical condition	-0.344	0.074	-0.201	-4.677*		
	Satisfaction with children's employment	-0.241	0.105	-0.108	-2.307*		
	Retirement	0.499	0.178	0.119	2.797*		
	Gender	-0.235	0.099	-0.101	-2.364*		
	Satisfaction with children's marriage	-0.187	0.091	-0.096	-2.070*		
Competence	Constant	5.782	0.400	-	14.454*	0.320	11.425*
	Monthly personal income (RMB)	0.307	0.068	0.197	4.515*		
	Physical condition	-0.368	0.083	-0.193	-4.425*		
	Gender	-0.334	0.112	-0.129	-2.974*		
	The number of children	-0.248	0.107	-0.098	-2.303*		
	Self-care	-0.574	0.253	-0.099	-2.271*		
Relatedness	Constant	7.597	0.300	-	25.327*	0.335	12.684*
	Satisfaction with children's personal income	-0.321	0.098	-0.150	-3.266*		
	Physical condition	-0.318	0.074	-0.186	-4.268*		
	Satisfaction with children's marriage	-0.242	0.088	-0.125	-2.735*		
	Gender	-0.267	0.099	-0.115	-2.699*		
	Self-care	-0.505	0.225	-0.097	-2.243*		
Total scale	Constant	6.523	0.318	-	20.533*	0.366	15.459*
	Physical condition	-0.339	0.064	-0.230	-5.328*		
	Satisfaction with children's personal income	-0.284	0.079	-0.153	-3.614*		
	Gender	-0.320	0.085	-0.160	-3.751*		
	Monthly personal income (RMB)	0.176	0.052	0.146	3.372*		
	Self-care	-0.439	0.193	-0.097	-2.279*		

* *P* < 0.05

### Autonomy

Results of multivariate analysis showed that there was no multicollinearity among the independent variables (tolerance 0.827 ~ 0.998, VIF 1.002 ~ 1.209). Empty-nesters of physical health well, children's employment and marriage satisfaction highly, retirement, and female have higher autonomy scores.

### Competence

Results of multivariate analysis showed that there was no multicollinearity among the independent variables (tolerance 0.940 ~ 0.987, VIF 1.013 ~ 1.064). Empty-nesters of personal monthly income highly, physical health well, female, two or more children, and partial self-care have higher competence scores.

### Relatedness

Results of multivariate analysis showed that there was no multicollinearity among the independent variables (tolerance 0.845–0.982, VIF 1.019–1.184). Empty-nesters of children's personal income and marriage satisfaction highly, physical health well, female, and partial self-care have higher relatedness scores.

### Total scale

Results of multivariate analysis showed that there was no multicollinearity between the independent variables (tolerance 0.925 ~ 0.960, VIF 1.041 ~ 1.082). Empty-nesters of physical health well, children's income satisfaction highly, female, personal monthly income highly, and partial self-care have higher total scale scores.

### Sociodemographic and children-related factors of multiple happiness

According to the MHQ, HI, PWB and SWB divided into dependent variables, and all socio-demographics are used as independent variables. Assignment is shown in Table 3. Multiple linear regression analysis results are shown in Table 5.

**Table 5:** Multiple linear regression analysis of influencing factors about multiple happiness in empty-nesters

Dependent variable	Independent variable	*B-value*	*S.E.-value*	*β-value*	*t-value*	*R-value*	*F-value*
Happiness index	Constant	8.742	0.212	-	41.164*	0.401	24.054*
	Physical condition	-0.566	0.090	-0.260	-6.281*		
	Satisfaction with children's personal income	-0.587	0.114	-0.215	-5.170*		
	Living status	-0.547	0.161	-0.139	-3.395*		
	The number of children	-0.288	0.120	-0.100	-2.412*		
Psychological well-being	Constant	33.393	1.284	-	26.017*	0.425	27.708*
	Physical condition	-2.551	0.359	-0.291	-7.112*		
	Monthly personal income (RMB)	1.686	0.297	0.236	5.682*		
	Gender	-1.717	0.493	-0.144	-3.486*		
	Satisfaction with children's marriage	-1.113	0.404	-0.112	-2.755*		
Subjective well-being	Constant	13.140	0.730	-	18.010*	0.502	24.045*
	Physical condition	-1.075	0.145	-0.299	-7.424*		
	Satisfaction with children's employment	-0.792	0.202	-0.168	-3.930*		
	Satisfaction with children's marriage	-0.649	0.174	-0.159	-3.722*		
	Gender	-0.829	0.197	-0.169	-4.216*		
	Monthly personal income (RMB)	0.366	0.118	0.124	3.100*		
	Self-care	-1.240	0.437	-0.113	-2.837*		
	Living status	-0.719	0.256	-0.111	-2.815*		

^*^ *P* < 0.05

### Happiness index

Results of multivariate analysis showed that there was no multicollinearity among the independent variables (tolerance 0.969 ~ 0.992, VIF 1.008 ~ 1.031). Empty-nesters of physical health well, children's personal income satisfaction highly, living with spouse, and two or more children have higher HI scores.

### Psychological well-being

Results of multivariate analysis showed that there was no multicollinearity among the independent variables (tolerance 0.948 ~ 0.983, VIF 1.017 ~ 1.055). Empty-nesters of physical health well, personal monthly income highly, female, and children's marital status satisfaction highly have higher PWB scores.

### Subjective well-being

Results of multivariate analysis showed that there was no multicollinearity among the independent variables (tolerance 0.818–0.968, VIF 1.033–1.223). Empty-nesters of physical health well, children's employment and marriage satisfaction highly, female, personal monthly income highly, partly self-care, and living with spouse have higher SWB scores.

### Correlation between basic psychological need satisfaction and multiple happiness

Correlation analysis results of BPNS and multiple happiness are shown in Table 6.

**Table 6:** Correlation analysis results

	Autonomy	Competence	Relatedness	Total scale	Happiness index	PWB	SWB
Autonomy	1						
Competence	0.541*						
Relatedness	0.564*	0.576*					
Total scale	0.816*	0.849*	0.840*	1			
Happiness index	0.411*	0.401*	0.430*	0.492*	1		
PWB	0.587*	0.624*	0.591*	0.710*	0.580*	1	
SWB	0.549*	0.560*	0.598*	0.679*	0.650*	0.785*	1

^*^ *P* < 0.05

### Prediction effect of basic psychological need satisfaction on multiple happiness

Predictive analysis results of BPNS on multiple happiness are shown in Table 7.

**Table 7:** Multiple linear regression analysis of basic psychological need satisfaction on multiple happiness

Dependent variable	Independent variable	*B-value*	*S.E.-value*	*β-value*	*t-value*	*R-value*	*F-value*
Happiness index	Constant	2.849	0.329	-	8.656*	0.484	51.308*
	Relatedness	0.321	0.063	0.252	5.062*		
	Competence	0.201	0.057	0.176	3.559*		
	Autonomy	0.192	0.062	0.151	3.110*		
PWB	Constant	8.841	1.064	-	8.312*	0.711	171.457*
	Competence	1.595	0.183	0.347	8.726*		
	Autonomy	1.408	0.199	0.276	7.074*		
	Relatedness	1.185	0.205	0.232	5.783*		
SWB	Constant	-0.136	0.467	-	-0.291^a^	0.661	129.907*
	Relatedness	0.698	0.090	0.331	7.756*		
	Competence	0.455	0.080	0.241	5.677*		
	Autonomy	0.461	0.087	0.220	5.281*		

^*^ *P* < 0.05; a: *P* = 0.771

### Model-fit index of structural equation model

**Fig. 2:**
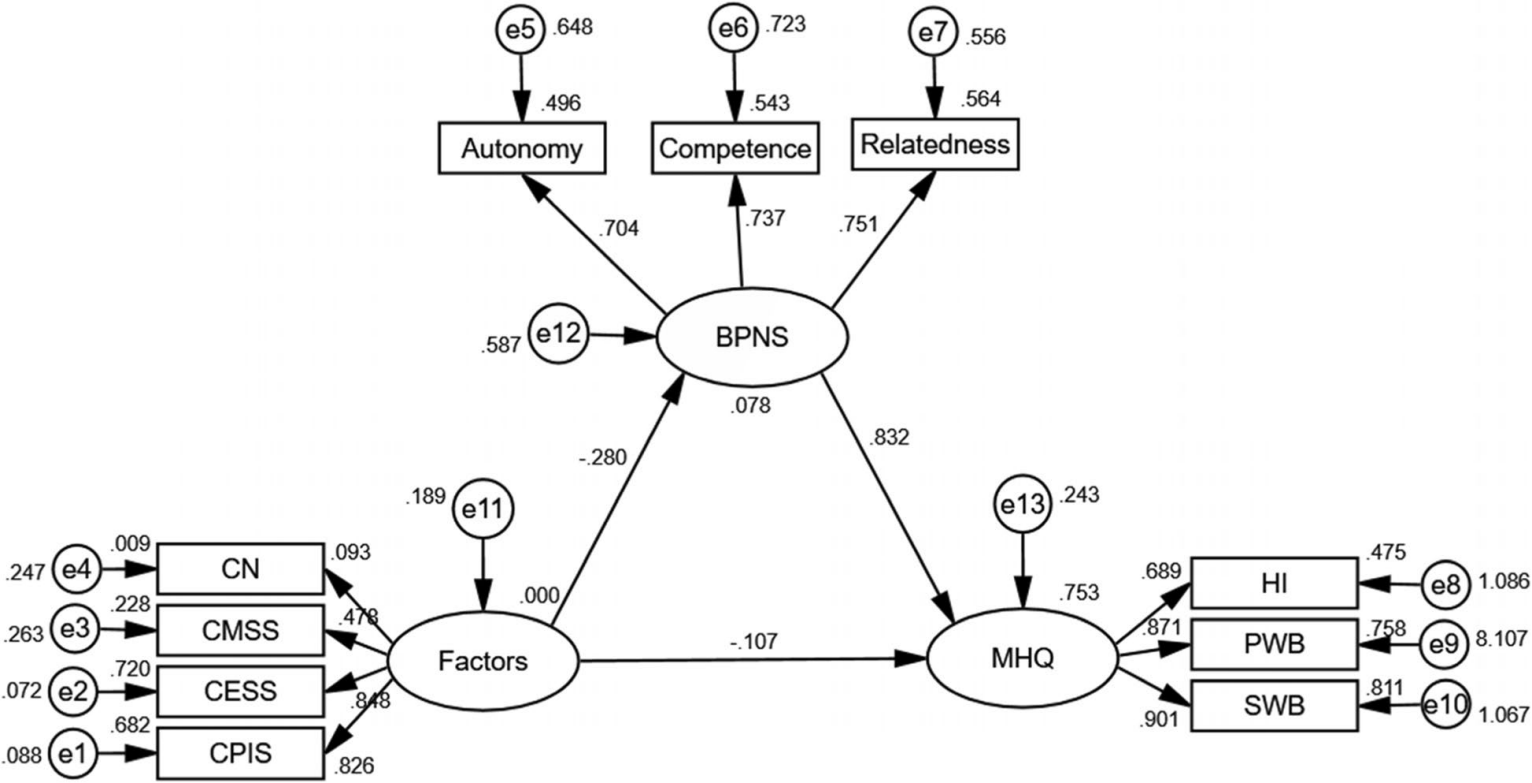
The structural equation model of multiple happiness among empty-nesters in Fuzhou, China Note. CN: Children's number, CMSS: Children's marital status satisfaction, CESS: Children's employment status satisfaction, CPIS: Children's personal income satisfaction

Structural equation modeling was employed for testing a hypothesized model for multiple happiness among the empty-nesters. The hypothesized model consisted of three latent factors (Fig. 2: Factors, BPNS, and MHQ) and ten observed variables. The observed variables were statistically significant in multiple linear regression. Three latent factors are measured by several observed indicator variables. It is worth mentioning that main and direct influencing factor of the happiness of empty-nesters is related to their adult children [22]. Therefore, this study only included four observation variables related to children (Children's number, Children's marital status satisfaction, Children's employment status satisfaction, and Children's personal income satisfaction) in SEM for analysis. Results are shown in Table 8, indicating that the hypothetical model provides a good fit for the data.

**Table 8:** Model-fit index of Confirmatory Factor Analysis(*n* = 507)

Inspected Fit Indices	Acceptable Fit	Confirmatory Factor Analysis Fit Indices	Degree of Fit
*χ*^*2*^/*df* (*χ*^*2*^ = 106.999, *df* = 32)	< 5.0	3.344	Well
SRMR	< 0.08	0.046	Well
RMSEA	< 0.08	0.068	Well
GFI	> 0.90	0.958	Well
AGFI	> 0.90	0.928	Well
CFI	> 0.90	0.964	Well
TLI	> 0.90	0.949	Well
NFI	> 0.90	0.950	Well

*χ*^*2*^/*df* = the ratio of the *X*^*2*^value and the degrees of freedom

Figure 2 illustrates the model results. Children-related factors had a direct effect on BPNS (*β* = -0.280, *P* < 0.001). BPNS had a direct effect to MHQ (*β* = 0.832, *P* < 0.001). Meanwhile, BPNS mediated the relationship between children-related factors and MHQ. The direct effect was -0.107, and the indirect effect was -0.233. Moreover, the greatest absolute value of standardized total effects was from BPNS (0.832), followed by children-related factors (0.340). Table 9 presented the total, direct and indirect effects of the variables on MHQ.

**Table 9:** Standardized total, direct and indirect effect of the variables on MHQ

Path	Total effect	Direct effect	Indirect effect
Factors → BPNS	-0.280	-0.280	0
BPNS → MHQ	0.832	0.832	0
Factors → MHQ	-0.340	-0.107	-0.233

## Discussion

### Status quo of basic psychological need satisfaction and multiple happiness

Three basic psychological needs satisfaction of autonomy, competence, and relatedness, and the overall level of urban empty-nesters in Fuzhou are high. Among them, relatedness is the highest, which is consistent with Tang [40], indicating that BPNS of empty-nesters is more reflected in relatedness. With age increasing, older adults slowly separate from their social roles and have less contact with the outside world and social networks shrink [54]. At the same time, the absence of children will make older adults who enter the empty-nest period lose social support and emotional communication. They would feel lonely, isolated, and even helpless due to self-doubt. The long-term effects of this harmful state could lead to empty nest syndrome, which reduces SWB [11]. It shows that we should pay more attention to interpersonal relationships and continuing social interactions of older adults.

In addition, this article measures multiple happiness of 507 empty-nesters living in coastal urban communities in eastern China. 73.57% of empty nesters' self-reported HI was above the middle level. PWB, SWB, and HI scores can also support above results that empty-nesters generally have higher happiness. Xi [55] found that total well-being of empty-nesters in China was at a higher level in 2020. Vast majority of empty-nesters in Fuzhou indeed had higher happiness scores than results measured by the same questionnaire in Nanchang [48], Hefei [56], and Henan [57], excepting negative effects. Empty-nesters' well-being in Fuzhou is higher than in other provinces and cities, relating likely to Fuzhou's economics, geographical location, ecological environment, and other factors.

The higher the BPNS, the greater the happiness [30]. BPNS and well-being of empty-nesters in Fuzhou are both at a high level. Is there any connection between them?

### Sociodemographic and children-related factors of basic psychological need satisfaction

Many factors influence the BPNS of empty-nesters. Firstly, empty-nesters with self-assessed physical health well have a higher BPNS. Self-assessed methods can reflect their physical and mental health [58]. Self-assessed health is good, indicating that physical and mental conditions are stable. Their psychological needs are more likely to be met. Secondly, women have a higher BPNS than men, which is inconsistent with Jiaojiao [59], who found that competence of men is higher than women, which may be caused by women's lack of self-confidence. In fact, women are less able to accumulate wealth across the life course due to a range of factors including lower education, more fragmented career progression, and lower income than men. Older women are also more likely to experience loss of a spouse, loneliness and social isolation, disability, and financial hardship [60]. Males seem to be generally more satisfied with their needs than females. However, our study presented the opposite conclusion, which reminds us that the reasons need to be further explored in future work. Thirdly, empty-nesters with higher personal monthly income have a higher BPNS. Stability and dominance of personal earning can promote empty-nesters completing in various affairs. Fourthly, Retired empty-nesters have higher autonomy, which may be related to retirees having more discretionary time. Fifth, the competence of parents with two or more children is higher than those with only one child, which is inconsistent with Zhilei [61]. Parents have entered the stage of being supported, and the parenting pressure of having children starts to translate into the advantage of having caregivers to raise parents [62]. Sixth, empty-nesters who are more satisfied with their children's employment, income, and marriage have higher BPNS. Children have a happy marriage and a harmonious family, which can improve their parents' spiritual satisfaction. In addition, the higher children's occupational status, stable jobs, and income levels, the higher their parents' spiritual satisfaction because those would directly influence their parents' quality of life and reduce life pressure [22]. Our research shows that the BPNS of empty-nesters who partially self-care is higher than that of completely independent people, which seems contrary to common sense. Among 507 participants, 482 older adults (95.10%) are fully self-care, and partially self-care (4.90%) is a small proportion, excluding those with poor self-care ability who cannot answer, thus affecting the analysis results. Therefore, we do not have sufficient evidence to support this claim.

### Sociodemographic and children-related factors of multiple happiness

Many factors influence the MHQ of empty-nesters. Firstly, empty-nesters with self-assessed physical health well have higher HI, PWB, and SWB. Physical health is the premise and basis for ensuring the quality of life [63]. Their good assessment indicates that older adults have high confidence and expectations in their health status and well-being [62]. Poor physical health can cause pain and psychological pressure and reduce well-being [64]. Secondly, empty-nesters who are more satisfied with their children's income, employment, and marriage have higher happiness. The income level of children is a reliable indicator for analyzing the self-rated happiness of their parents [22]. The higher the income and occupational rank of children, the higher the life satisfaction of parents [65]. It may be because the children have stable jobs and high incomes, which can reduce the financial burden of older adults to a certain extent and resolve the contradiction of gnawing the old. These increase the self-esteem and self-confidence of older adults, and their happiness increases accordingly. In addition, older adults with high satisfaction with their children's marital status have a lower detection rate of depressive symptoms and better mental health and well-being [66]. Thirdly, empty-nesters living with a spouse have higher HI, and SWB. The company of spouses is consolation for empty-nesters whose children are not around. Empty-nesters receive social support and life care, which effectively relieves their psychological pressure [67]. In addition, the absence of a spouse will aggravate the loneliness. There is also a lack of trustworthy objects for daily communication and emotional venting [55]. Fourthly, empty-nesters with higher monthly incomes have higher PWB and SWB. Economic status is the most significant factor affecting the well-being of empty-nesters [68]. Empty-nesters gain security, self-affirmation, and self-esteem and increase their well-being because their economic level is guaranteed [69]. Fifth, women have higher PWB and SWB than men, which is inconsistent with previous research [70, 71]. The possible reason is that men have a wider social field than women and are more likely to find a sense of group belonging. While women have been family-centered since ancient times, the negative emotions caused by family conflicts will decrease happiness. Sixth, the HI of parents with two or more children is higher than those with only one child. The possible reason is that the more children there are, the more resources older adults got in their daily life, including economic resources and social support. Our research shows that the SWB of empty-nesters who partially self-care is higher than that of completely independent people, which seems contrary to common sense. Among 507 participants, 482 older adults (95.10%) are fully self-care, and partially self-care (4.90%) is a small proportion, excluding those with poor self-care ability who cannot answer, thus affecting the analysis results. Therefore, we do not have sufficient evidence to support this claim.

### Relationships between children-related factors, basic psychological need satisfaction, and multiple happiness

We explore the association between children-related factors, BPNS, and MHQ, and verified the relationship among them. It is worth mentioning that this study is the first to confirm that BPNS can positively predict HI, PWB, and SWB simultaneously among empty-nesters in southern China. Structural equation model results showed that BPNS directly impacted MHQ, and children-related factors indirectly affect MHQ through BPNS. This study provides new insight into the underlying mechanism of happiness of empty-nesters and its influencing factors.

According to the structural equation modeling, BPNS made directly impacted MHQ. This result was consistent with older adults in Beijing (northern China) [40] or European and American countries [41], indicating that whether in European and American countries or Asian countries, satisfying basic psychological needs could directly and positively affect happiness. The reason is meeting needs are associated with more secure attachment, better relationship quality, less perceived conflict, and more adaptive responses to conflict [72]. In addition, Ryan and Deci [28] thought that fulfilling basic psychological needs are the source of individual PWB. Tay and Diener [73] pointed out that need fulfillment was consistently associated with SWB, which is also one of the predictors of psychological needs satisfaction. This study has the same conclusion as the above studies and believes that three basic psychological needs could predict PWB, SWB, and HI together and enriches the applicability of Self-Determination Theory to the happiness of empty-nesters in southern China.

Another finding was the mediating role of BPNS in the relationship between children-related factors and MHQ. Empty-nesters with many children and high satisfaction with children's marriage, work, and income had better happiness [65, 66]. With increasing age, children are one of the most significant emotional sustenance for empty-nesters [74]. For the Chinese, the family relationship is closely related to relieving psychological distress through emotional sustenance and is a power source of social support [54]. A harmonious family relationship means they have more opportunities to communicate with their children, thus getting more help and maintaining good mental health [75]. Family support has a critical impact on the health of empty-nesters. Therefore, we should encourage our children to spend more time with the empty-nesters and communicate with them, which could help them relieve their negative emotions and improve their well-being.

### Limitations

This study had some limitations. First, we used cross-sectional data, so it cannot offer solid causal conclusions. Thus, researchers should explain the results of this study prudently. Future research based on longitudinal data or randomized controlled trials is still needed. Second, the study contained only some common demographic characteristics. Other variables of respondents, such as pension models, living arrangements, and religious beliefs are not analyzed. Simultaneously, our research considers only two aspects of children-related factors. Other relevant variables were also not explored. For example, children help their parents do housework or provide emotional comfort. Future studies could consider analyzing them to explore a more intricate relationship between BPNS and MHQ. Third, the study provides an efficient reference value for older adults living in southern coastal cities, but data is limited for those in rural or inland regions. Fourth, this study does not focus on a comparison between empty-nesters and non-empty-nesters. Future studies can increase comparison between them to highlight the necessity to study the influencing factors of BPNS and MHQ in empty-nesters.

## Supplementary Information

**Additional file 1. **Participants’ raw data.

## Data Availability

The raw data have been put into the Supplementary Information file. The datasets generated and/or analyzed during the current study are not publicly available because the raw data comes from the first author’s master’s project. The first author Yang Yu-ting’s graduation thesis has a confidentiality period of 2 years, and other related papers have not yet been published. But the raw data are available from the corresponding author on reasonable request.
